# The complete mitochondrial genome of sponge *Tethya* sp. (Demospongiae, Tethyida, Tethyidae)

**DOI:** 10.1080/23802359.2016.1186518

**Published:** 2016-07-10

**Authors:** Yuan Zhang, Dan Huang, Dexiang Wang, Shaoxiong Ding

**Affiliations:** a Fujian Provincial Key Laboratory for Coastal Ecology and Environmental Studies, Xiamen University, Xiamen, China;; b Marine Biodiversity and Global Change Research Center, Xiamen University, Xiamen, China

**Keywords:** Demosponge, mitogenome, sponge, *Tethya* sp.

## Abstract

The complete mitochondrial genome of *Tethya* sp. was studied. This is the second complete mitochondrial report on the family Tethyidae. The mitochondrial genome of *Tethya* sp. is 20,582 bp in length, containing 14 protein-coding genes and 25 tRNA genes, with 2 rRNA genes. Our phylogenetic result suggested that *Tethya* sp. converged well with *Tethya actinia*, which further verified the morphological result. We anticipate our study to shed light on future molecular studies of demosponges.

Sponge systematics is a long-standing issue due to vulnerability to environmental modification which enhancing the difficulty in taxonomy. As a supplementary measure for morphological taxonomy, studies have applied the mitochondrial genome of metazoa as a marker to resolve taxonomic controversies (Gissi et al. 2008). Little information is known about the evolution of sponges. Currently, only one complete mitogenome of *Tethya actinia* has been reported for the genus *Tethya* (Lavrov et al. 2005). In this study, we concentrated on exploring an approach of aligning mitochondrial genome with other demosponges to describe the molecular relationships among them.

*Tethya* sp. was sampled from Gulei Peninsula, Fujian Province, China (117.5928E 23.8015N), in May 2014 and deposited in the Museum of Marine Science and Technology, Xiamen University with the number XMU02001 079. DNA was extracted using guanidinium isothiocyanate with the method adapted from Wilson and Carson (2001) and sequenced with MPS (massive parallel sequencing) Illumina technology. The sample was constructed in a paired-end library with an insert size of 420 bp and was sequenced using a Hiseq2500 (Beijing, China) by PE125 strategy. Sequencing and annotation was performed at Beijing Novogene Bioinformatics Technology Co. Ltd. Clean reads were assembled by SOAPdenovo (Li et al. 2008, 2010) to produce a single circular form of the complete mitochondrial genome. A whole genome Blast (Altschul et al. 1990) search (E-value ≤1e − 5, minimal alignment length percentage≥ 40%) against 6 databases, KEGG (Kanehisa 1997; Kanehisa et al. 2004; Kanehisa et al. 2006), COG (Tatusov et al. 1997; Tatusov et al. 2003), NR, Swiss-Prot (Magrane & Consortium 2011), GO (Ashburner et al. 2000) and TrEMBL (Magrane & Consortium 2011), was conducted. An alignment of the assembled scaffold for *Tethya* sp. demonstrated that mitogenome of *Tethya actinia* (GenBank number: AY320033.1) shared many similarities (86% identity) with our sample. We took the homological mitogenome as reference using the mitochondrial genome annotation (MITOS) server (Bernt et al. 2013) for annotation and BLASTX to improve results. Subsequently, we obtained annotated coding DNA sequences (CDS), transfer RNA genes (tRNA) and ribosomal RNA (rRNA) genes.

The complete genome of *Tethya* sp. is 20,582 bp long with 14 protein-coding genes, 25 tRNA genes and 2 rRNA subunits. The base composition of the mitogenome is A (29.79%), T (35.22%), C (11.93%), and G (23.07%), with a GC content of 35.38% (Table 1). The annotated mitogenome has been submitted to NCBI (GenBank accession number KU748128).

**Table 1.** Mitochondrial genome organization of *Tethya* sp.

Name	From	To	Direction	Length (bp)
*trnF* (gaa)	35	107	+	73
*rrnS*	111	1363	+	1253
*trnG* (tcc)	1458	1529	+	72
*trnV* (tac)	1648	1719	+	72
*rrnL*	1739	4392	+	2654
*trnE* (ttc)	4505	4576	+	72
*nad6*	4580	5131	+	552
*trnY* (gta)	5161	5232	+	72
*trnM* (cat)	5300	5371	+	72
*cox2*	5424	6086	+	663
*trnL* (taa)	6134	6216	+	83
*trnK* (ttt)	6313	6384	+	72
*atp8*	6447	6599	+	153
*atp6*	6897	7418	+	522
*trnR* (tct)	7463	7535	+	73
*cox3*	7548	9330	+	783
*trnN* (gtt)	8361	8432	+	72
*cob*	8520	9653	+	1134
*trnS* (gct)	9682	9755	+	74
*trnI* (gat)	9789	9861	+	73
*trnQ* (ttg)	9915	9986	+	72
*trnW* (tca)	10,036	10,106	+	71
*atp9*	10,245	10,478	+	234
*nad4*	10,675	11,958	+	1284
*trnH* (gtg)	12,048	12,120	+	73
*trnD* (gtc)	12,159	12,230	+	72
*nad3*	12,334	12,663	+	330
*trnR* (tcg)	12,797	12,867	+	71
*nad4l*	12,907	13,158	+	252
*cox1*	13,299	14,840	+	1542
*trnS* (tga)	14,991	15,071	+	81
*nad1*	15,187	16,149	+	963
*trnP* (tgg)	16,230	16,302	+	73
*trnL* (tag)	16,463	16,536	+	74
*trnC* (gca)	16,602	16,673	+	72
*trnT* (tgt)	16,685	16,757	+	73
*trnM* (cat)	16,840	16,910	+	71
*nad2*	17,268	18,179	+	912
*nad5*	18,522	20,282	+	1761
*trnA* (tgc)	20,322	20,394	+	73
*trnM* (cat)	20,445	20,516	+	72

Direction: “+” stands for 5′ to 3′, “−” stands for 3′ to 5′.

In *Tethya* sp., the arrangement of protein-coding genes and gene contents was completely identical to that of *T. actinia*. After multiple sequence alignment with other mitogenomes of demosponges by MAFFT (Katoh & Standley 2013), we constructed a phylogenetic tree (Figure 1) using Maximum Likelihood (ML) method in MEGA 6.06 (Tamura et al. 2013). The phylogenetic result indicated that *Tethya* sp. converged well with *Tethya actinia*, which further verified the morphological result. In conclusion, mitogenome studies of sponges may contribute to a better phylogenetic understanding of demosponges, subsequently influencing the knowledge of molecular evolution of metazoa.

**Figure 1.**
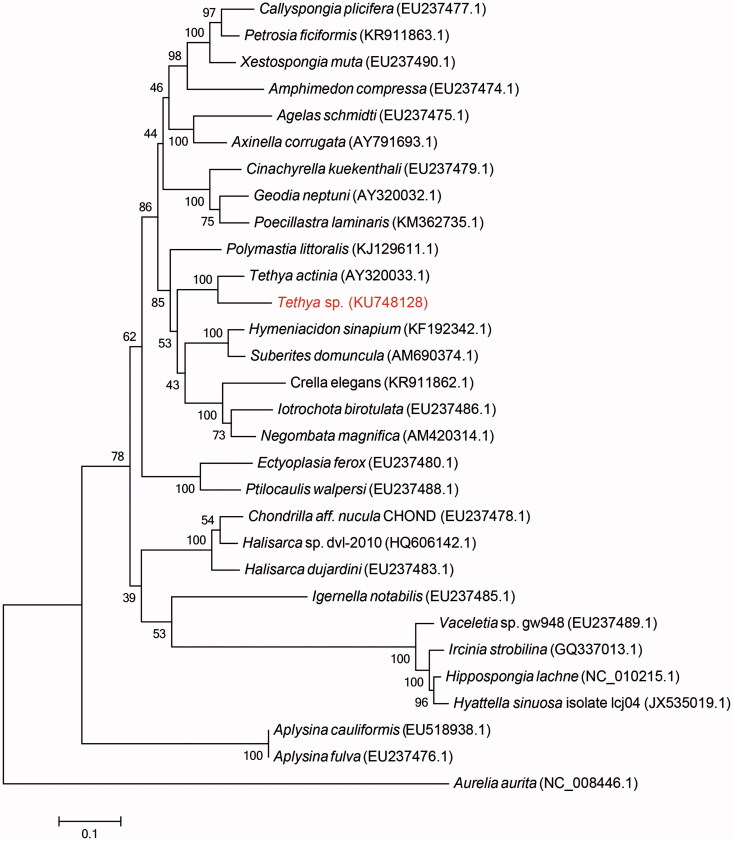
The consensus phylogenetic relationship of *Tethya* sp. and the other species of Demospongiae as well as *Aurelia aurita* (a kind of jellyfish) from Maximum Likelihood (ML) analysis with 1000 bootstrap. The number on the branches are the bootstrap values for ML. The Genbank accession numbers of each species are shown in the brackets.
